# Impact of probiotic supplementation on salivary function, oral microbiota, and gut health: a systematic review

**DOI:** 10.3389/fcimb.2026.1816010

**Published:** 2026-06-04

**Authors:** Gunjan Kumar, Samikshya Jena, Vinod Kumar Nelson, Habeeb Ali Baig, Zaid Alkhalfi Alanazi, Bader Khalid Alanazi, Fahad Saad Z. Alanazi, Alenazi Ahmad Awad R

**Affiliations:** 1 Department of Public Health Dentistry, Kalinga Institute of Dental Sciences (KIDS), Kalinga Institute of Industrial Technology (KIIT) Deemed to be University, Bhubaneswar, Odisha, India; 2 Department of Pharmaceutical Chemistry, Mahathi College of Pharmacy, Madanapalli, Andhra Pradesh, India; 3 Department of Microbiology, Faculty of Medicine, Northern Border University, Arar, Saudi Arabia; 4 Department of Family Medicine, Faculty of Medicine, Northern Border University, Arar, Saudi Arabia; 5 Department of Internal Medicine, Faculty of Medicine, Northern Border University, Arar, Saudi Arabia; 6 Department of Family and Community Medicine, Faculty of Medicine, Northern Border University, Arar, Saudi Arabia; 7 Family Medicine Senior Registrar, National Guard Health Affairs, Northern Border Province, Arar, Saudi Arabia

**Keywords:** gut microbiome, oral microbiota, probiotic supplementation, randomized controlled trials, salivary parameter

## Abstract

**Introduction:**

Probiotics, which are classified as helpful living microorganisms, have demonstrated the ability to improve salivary function, inhibit pathogens like Streptococcus mutans, and modify the oral and gut microbiota. The aims of this systematic review was to assess their effects on salivary function, oral microbiota, and gut health.

**Methods:**

Literature search was done in PubMed, Scopus, Web of Science, Science Direct and Cochrane were performed. Randomized controlled trials were included which involved human subjects receiving probiotic supplementation Outcomes assessed were salivary parameters (flow rate, buffering capacity, pH, biomarkers), oral microbiota changes, and gut health indicators. Study selection, data extraction, and risk of bias assessment (RoB 2.0) were performed independently by two reviewers. Due to heterogeneity, meta-analysis was not conducted.

**Results:**

A total of six systematic reviews were included in this review. Probiotic supplementation was associated with improvements in salivary parameters, including buffering capacity and plaque pH, and reductions in cariogenic bacteria such as *Streptococcus mutans*. Probiotics demonstrated beneficial effects on gut microbiota and gastrointestinal symptoms, supporting an oral–gut microbiota interaction. Risk of bias ranged from low to high across studies. Overall, evidence suggested beneficial effects, though heterogeneity and methodological limitations reduced certainty.

**Conclusions:**

Probiotics have been shown to provide modest, strain-specific benefits for modulating the oral microbiota and certain salivary parameters, with more precise molecular and clinical evidence for gut effects.

**Systematic Review Registration:**

https://www.crd.york.ac.uk/PROSPERO/view/CRD420251243347, identifier CRD420251243347.

## Introduction

The oral cavity is one of the body’s most varied microbial ecosystems, and the human microbiome is essential for preserving both dental and systemic health (Lin et al., 2022). An essential part of maintaining oral homeostasis, saliva aids in dental tissue lubrication, buffering ability, antibacterial defense, and remineralization (Hemarajata and Versalovic, 2013). Common oral diseases such dental caries, periodontal disease, and oral mucosal disorders are closely linked to changes in salivary function and oral microbial balance (de Oliveira et al., 2022).

In recent years, probiotics defined by the Food and Agriculture Organization and the World Health Organization as live microorganisms that, when ingested in sufficient quantities, offer the host quantifiable health advantages have gained increasing attention as a potential adjunctive strategy for promoting oral and gut health (Arweiler et al., 2020). The gastrointestinal system has historically been the focus of this field’s research, and there is strong evidence that probiotics can improve gut microbial profiles, maintain the integrity of the intestinal barrier, control immune function, and reduce the frequency of diarrhea caused by antibiotics (Hemarajata and Versalovic, 2013). More recently, focus has shifted to the oral environment because mounting evidence suggests probiotic therapies may improve oral health by directly influencing oral microbial communities and by having systemic effects that affect oral tissues (Salim et al., 2023).

Probiotics reach the human body through the mouth cavity, which is home to a highly varied microbial environment with over 700 species organized into organized biofilms (Babina et al., 2023). Saliva is essential for maintaining oral homeostasis because it helps regulate pH, provides immune-active and antibacterial components, promotes enamel remineralization, and restricts microbial attachment to oral surfaces (Babina et al., 2023). Dental caries, oral infections, malodor, and periodontal disease may become more likely if salivary production, buffering capacity, or biochemical composition are disrupted. Determining whether probiotic supplementation can favorably alter salivary parameters, such as flow rate, buffering capacity, enzymatic activities, and indicators of oral inflammation, has drawn increasing attention in this context (Mo et al., 2022).

The effects of specific probiotic strains, including Lactobacillus reuteri, Lactobacillus rhamnosus, and different Bifidobacterium species, on salivary features and oral microbial composition have been investigated in a number of human clinical investigations. The results of these studies suggest that the administration of probiotics may influence salivary inflammatory mediators, change the profile of periodontal pathogens, and reduce the quantity of cariogenic microorganisms such Streptococcus mutans (Lin et al., 2022). The biological processes that are thought to be responsible for these effects include competition with pathogenic bacteria for nutrients and adhesion sites, secretion of antimicrobial substances like organic acids and bacteriocins (including reuterin), interference with the formation of dental biofilms, and control of host immune activity at the oral mucosal level (Sulaiman et al., 2024). Nevertheless, reported results vary amongst studies, which could be explained by differences in the choice of probiotic strains, dosages given, delivery methods (such as lozenges, dairy matrices, or capsules), duration of supplementation, and techniques for assessing clinical and microbiological endpoints (Koopaie et al., 2019).

Probiotics may impact oral health through systemic routes by altering the gut microbiota in addition to their direct effects within the mouth cavity, highlighting the growing interest in the oral-gut axis (Nadelman et al., 2018). A growing body of evidence suggests a reciprocal link between oral and intestinal microbial populations, with gut-mediated immunological and inflammatory signals affecting oral tissues and germs originating in the oral cavity influencing gut ecology. Probiotics can alter gut microbial profiles, stimulate the synthesis of short-chain fatty acids, modify mucosal immune responses, and reduce systemic inflammation when taken orally. Salivary gland function and oral inflammatory state both improve as a result of these systemic impacts (de Oliveira et al., 2022).

Although research on probiotic medicines has increased dramatically, the information regarding their impact on gut health, oral microbial composition, and salivary function remains sparse and contradictory. Previous systematic reviews that have primarily examined probiotic use in oral health in a generic way have made limited attempts to simultaneously evaluate salivary results, oral microbiota dynamics, and gastrointestinal health within a single analytical framework. Additionally, variations in methodological approaches, brief intervention durations, limited study populations, and heterogeneity in outcome assessment instruments hinder the establishment of sound, evidence-based recommendations for the use of probiotics in dental care.

Therefore, this systematic review aims to critically evaluate and synthesize the available evidence on the effects of probiotic supplementation on salivary function, oral microbiota composition, and gut health. By integrating findings across these domains, this review seeks to provide a holistic understanding of the potential role of probiotics in promoting oral–systemic health and to identify gaps for future research.

## Methodology

### Study design and reporting standards

This systematic review was prospectively registered in the PROSPERO database (registration number: CRD420251243347) and conducted in accordance with the Preferred Reporting Items for Systematic Reviews and Meta-Analyses (PRISMA) 2020 criteria.

### Eligibility criteria (PICOS framework)

- Population (P):
  Human participants of any age group, including children, adolescents, adults, and older adults. Both healthy individuals and those with oral health conditions such as dental caries, gingivitis, periodontitis, or xerostomia were included.

- Intervention (I):
  Administration of probiotic supplementation in the form of single-strain or multi-strain formulations. Probiotics delivered through any vehicle, including lozenges, tablets, capsules, dairy products, or fortified foods. Only studies clearly reporting probiotic strain(s), dosage, and duration were considered.

- Comparison (C):
  Placebo, no intervention, standard care, or baseline measurements in before–after study designs.

- Outcomes (O):
  Studies reporting at least one of the following outcomes were included:

o Salivary function outcomes: salivary flow rate (stimulated or unstimulated), buffering capacity, salivary pH, enzymes, or inflammatory biomarkers.
o Oral microbiota outcomes: changes in oral microbial composition, counts of cariogenic or periodontal pathogens (e.g., *Streptococcus mutans*), or microbial diversity indices.
o Gut health outcomes: gut microbiota composition, gastrointestinal symptoms, and immune or inflammatory markers related to gut function.

- Study design (S):
  Randomized controlled trials (RCTs).

### Exclusion criteria

- Animal or *in vitro* studies,
- Case reports, case series, editorials, letters, or conference abstracts
- Studies not reporting oral, salivary, or gut-related outcomes
- Publications in languages other than English

### Search strategy

A systematic literature search was conducted to find studies that examined the effects of probiotic supplementation on salivary function, oral microbiota, and gut health. From the beginning until March 30, 2026, electronic databases such as PubMed/MEDLINE, PubMed Central (PMC), Scopus, Web of Science, ScienceDirect, and Cochrane Library were searched. Only English-language research with human subjects was included in the search.

Search terms were developed using Medical Subject Headings (MeSH) and free-text keywords related to probiotics (e.g., probiotic, *Lactobacillus*, *Bifidobacterium*, *Limosilactobacillus reuteri*), salivary outcomes (e.g., saliva, salivary flow, buffering capacity, xerostomia), oral microbiota (e.g., oral microbiome, *Streptococcus mutans*, periodontal pathogens), and gut health (e.g., gut microbiota, intestinal microbiota). These were combined using Boolean operators (AND, OR).

To minimize publication bias, reference lists of included studies and relevant reviews were manually screened. Duplicate records were removed, and all retrieved citations were managed using reference management software. The detailed search strings are provided in the **Table 1**.

**Table 1:** Database search using Boolean operators.

PubMed-MEDLINE	(((“Probiotics [MeSH Terms] OR probiotics [tiab] OR Lactobacillus [MeSH] OR Lactobacillus[tiab] OR Bifidobacterium[MeSH] OR Bifidobacterium[tiab] OR “Streptococcus salivarius”[tiab] OR “beneficial bacteria”) AND [All Fields]Saliva[MeSH] OR saliva[tiab] OR “salivary glands”[MeSH] OR “salivary gland”[tiab] OR “salivary flow”[tiab] OR “salivary secretion”[tiab] OR “salivary biomarkers”[tiab]) AND (““Mouth microbiome”[MeSH] OR “oral microbiome”[tiab] OR “oral microbiota”[tiab] OR “oral flora”[tiab] AND “Gastrointestinal microbiome”[MeSH] OR “gut microbiome”[tiab] OR microbiome[tiab] OR dysbiosis[tiab])
Scopus	(TITLE-ABS-KEY (probiotic* OR lactobacillus OR bifidobacterium OR “streptococcus salivarius”)) AND (TITLE-ABS-KEY (saliva OR “salivary flow” OR “salivary gland” OR “salivary biomarkers”)) AND (TITLE-ABS-KEY (“oral microbiome” OR “oral microbiota” OR “oral flora” OR “mouth microbiome”)) AND (TITLE-ABS-KEY (“gut health” OR “gut microbiome” OR “gastrointestinal microbiome” OR dysbiosis))
Web of Science	TS= (probiotic* OR lactobacillus OR bifidobacterium OR “streptococcus salivarius”) AND TS=(saliva OR “salivary flow” OR “salivary gland*” OR “salivary biomarkers”) AND TS= (“oral microbiome” OR “oral microbiota” OR “oral flora” OR “mouth microbiome”) AND TS=(“gut health” OR “gastrointestinal microbiome” OR “gut microbiota” OR dysbiosis)
Science direct	“Probiotic” AND (saliva” OR salivary flow) AND (“oral microbiota” OR “oral microbiome” OR “oral flora “) AND “gastrointestinal health” OR “gut microbiota” OR “dysbiosis”)
Cochrane	Probiotic AND (saliva OR salivary flow) AND (oral microbiota OR oral microbiome OR oral flora) AND gastrointestinal health OR gut microbiota OR dysbiosis)

### Study selection process

**Figure 1:**
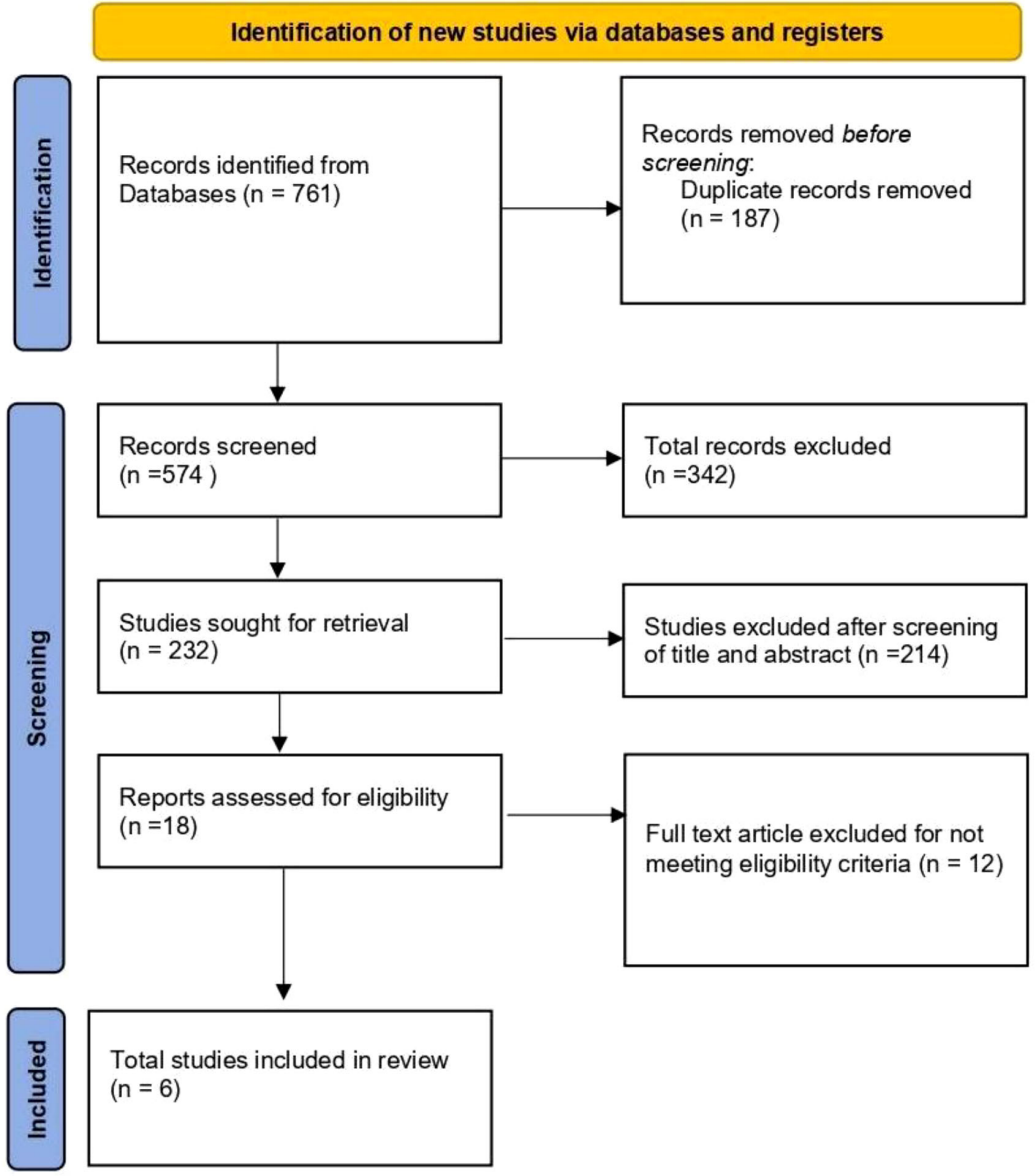
Flow chart summarizing the article selection process.

All identified records were imported into a reference management software, and duplicate records were removed. Two reviewers independently screened titles and abstracts for eligibility. Studies deemed potentially relevant were retrieved for full-text assessment. Full-text articles were evaluated independently by both reviewers against the predefined inclusion and exclusion criteria. Any disagreements were resolved through discussion and consensus. When necessary, a third reviewer was consulted to resolve discrepancies. The study selection process is summarized using a PRISMA 2020 flow diagram (Shown in **Figure 1**).

### Data extraction

Using a standardized data extraction form, two reviewers independently extracted the data. Each included study was analyzed to extract the following data: participant characteristics, study design and sample size, country of study, author and year of publication, probiotic strain or strains, dosage, formulation, and duration comparator or control intervention, primary and secondary outcomes related to salivary function, oral microbiota, and gut health, key findings and conclusions. Any discrepancies in data extraction were resolved through discussion.

### Risk of bias assessment

**Figure 2:**
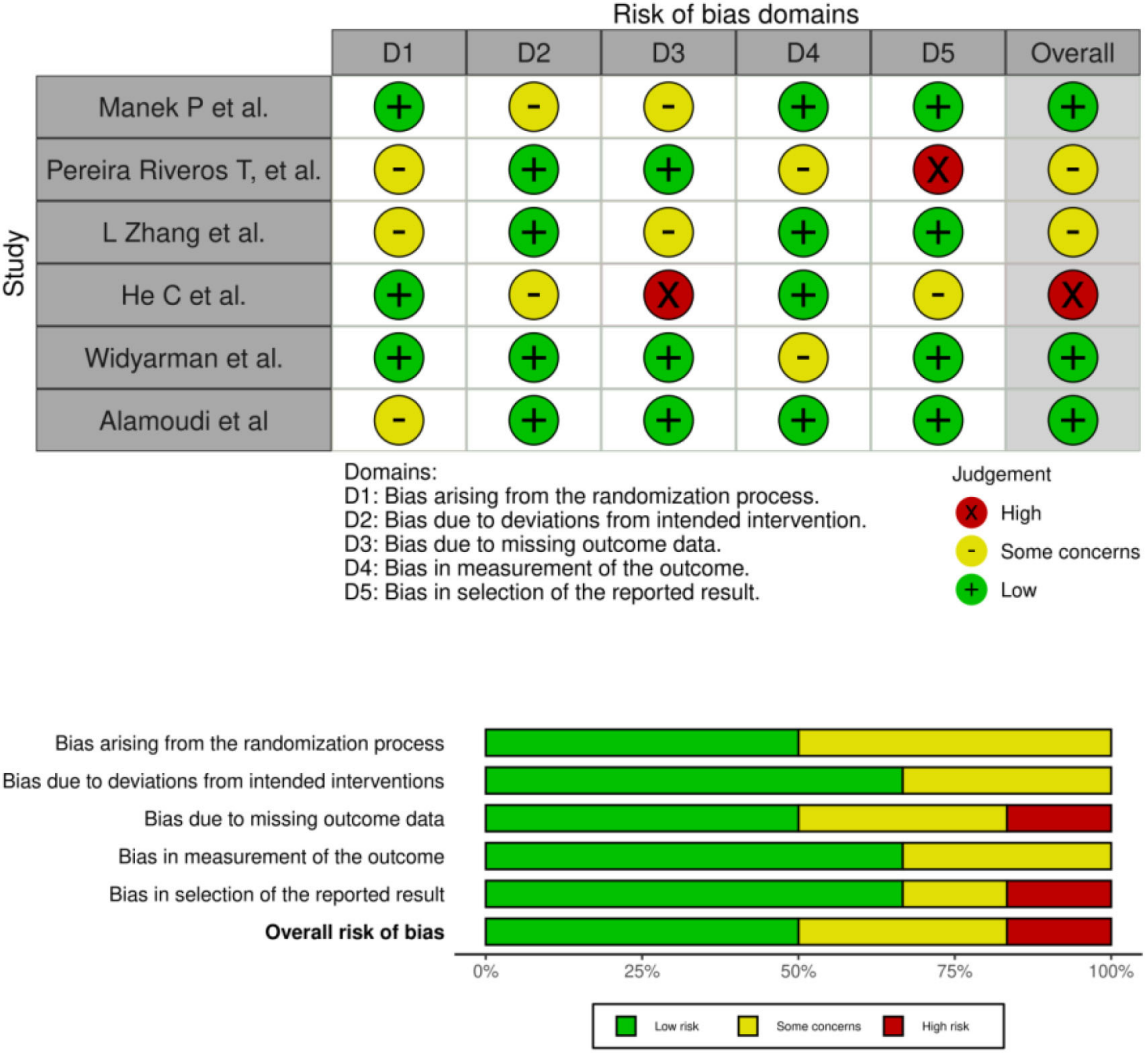
Quality assessment of the studies.

The Cochrane Risk of Bias instrument (RoB 2.0) was used to assess the internal validity of the included randomized controlled trials, as illustrated in **Figure 2**. Assessment was performed across five key domains: (i) potential bias related to the randomization process; (ii) bias arising from departures from the intended interventions; (iii) bias associated with incomplete or missing outcome data; (iv) bias in outcome measurement; and (v) selective reporting of results. Each domain was categorized as presenting a low risk of bias, some concerns, or a high risk of bias, which collectively informed the overall risk-of-bias judgment for each trial. Systematic reviews considered for background and contextual interpretation were not evaluated using the RoB 2.0 tool; however, their methodological rigor was examined through critical appraisal.

### Data synthesis

The results of the included research were interpreted and integrated using a narrative synthesis approach. A quantitative meta-analysis was first contemplated, but it was not possible because of significant variation among studies about probiotic strains, dosages, delivery methods, research populations, outcome measures, and follow-up periods. Furthermore, significant statistical pooling was further hampered by the small number of research within particular outcome areas.

Oral microbiota composition, salivary characteristics (such as flow rate, buffering capacity, and biochemical indicators), and gut microbiota and systemic impacts were the three main outcome categories into which the research were divided to guarantee clarity. Each category’s results were closely examined to find recurring patterns, parallels, and differences. The origins of variability, such as variations in probiotic properties and research design, were investigated using a critical interpretive approach. With consideration for methodological quality and bias risk, the general direction and strength of the evidence were evaluated qualitatively. This methodical narrative approach acknowledged inherent constraints while enabling a thorough and clear synthesis.

### Certainty of evidence

Based on study design, consistency of findings, bias risk, and directness of evidence, the overall certainty of the evidence was evaluated qualitatively using GRADE assessment tool (Shown in **Table 2**).

**Table 2:** GRADE assessment.

Outcomes	No. of participants (studies)	Study design	Risk of bias	Inconsistency	Indirectness	Imprecision	Other considerations	Effect	Certainty of evidence
Reduction in cariogenic bacteria (Alamoudi et al., 2018)	178 (1 study)	Randomized controlled trial	Not serious	Not serious	Not serious	Serious	None	Significant reduction in bacterial counts	⊕⊕⊕◯ MODERATE
Salivary flow & microbial improvement (Pereira Riveros et al., 2025)	61 (1 study)	Randomized controlled trial	Not serious	Not serious	Not serious	Serious	Small sample size	Increased salivary flow, reduced pathogens	⊕⊕⊕◯ MODERATE
Oral microbiome & hygiene indices (Widyarman et al., 2022)	40 (1 study)	Randomized controlled trial	Serious	Not serious	Not serious	Serious	No control group	Reduced pathogenic bacteria, improved OHI & PBI	⊕⊕◯◯ LOW
Plaque pH & gingival health (V Manek et al., 2025)	60 (1 study)	Randomized controlled trial	Serious	Not serious	Not serious	Serious	Small sample	Increased plaque pH, reduced gingival inflammation	⊕⊕◯◯ LOW
GI microbiota & adverse events (He et al., 2022)	276 (1 study)	Double-blind Randomized controlled trial	Not serious	Not serious	Not serious	Not serious	None	Reduced adverse events, microbiota stabilization	⊕⊕⊕⊕ HIGH
Immune & metabolic outcomes (Zheng et al., 2023)	140 (2 trials)	Randomized controlled trial	Not serious	Not serious	Serious	Serious	Indirect outcome	Improved immune response & inflammation	⊕⊕◯◯ LOW

## Results

The effects of probiotic supplementation on salivary function, oral microbiota, and associated oral or gut health outcomes were assessed in six original human clinical investigations. (Shown in **Table 3**) This analysis comprised six randomized controlled studies involving both healthy individuals and clinical populations that were carried out between 2018 and 2025 in various geographic locations (India, Brazil, China, Indonesia, and Saudi Arabia). Preschoolers, radiation patients, and healthy adults made up the sample sizes, which varied from 30 to 276.

**Table 3:** Result summary of the included study.

Authors	Year	Country	Study design	Population(n)	Probiotic strain(s)	Form/dose/duration	Outcomes evaluated	Main findings
V Manek et al. (2025)	2025	India	Randomized controlled clinical trial	Healthy adults (n = 60)	Multi-strain probiotic (Lactobacillus spp., Bifidobacterium spp.)	Oral supplement/capsules, once daily, 30 days	Plaque pH, Gingival index	Significant increase in plaque pH and significant reduction in inflammation compared with control
Pereira Riveros et al. (2025)	2025	Brazil	Randomized, double-blind, placebo-controlled	Head & neck cancer patients post-radiotherapy (n = 61 completed)	Multi-strain probiotic	Sachets, Daily, 30 days	Stimulated/unstimulated salivary flow, pH, total bacterial load, F. nucleatum counts	Increased stimulated salivary flow; reduced total bacterial load and F. nucleatum counts in saliva and culture reduced key pathogens compared to placebo
Zheng et al. (2023)	2023	China	Randomized controlled trial	Adults (n = 140)	Multi-strain probiotic	Capsules, 4 weeks	Gut symptoms, immune markers	Improved bowel habits and inflammatory markers
He et al. (2022)	2022	China	Randomized controlled trial	Adults (n=276)	Multi-strain probiotic	Capsules, 28 days	Gut symptoms, gut microbiota, oral microbiota	Reduced GI adverse effects and stabilized gut & oral microbiota
Widyarman et al. (2022)	2022	Indonesia	Randomized clinical trial	Healthy adults (n=30)	Lactobacillus reuteri	Oral lozenge, daily,2 weeks	Salivary microbiome composition	Short-term alteration of salivary microbiota; effects not sustained post-intervention
Alamoudi et al. (2018)	2018	Saudi Arabia	Randomized controlled trial	Preschool children (n=44)	Lactobacillus reuteri DSM 17938	Lozenges, twice daily, 4 weeks	Salivary buffering capacity, *S. mutans* counts	Significant reduction in *S. mutans* levels and improvement in salivary buffering capacity

The majority of studies examined multi-strain probiotic formulations that included Lactobacillus and Bifidobacterium species and were given as lozenges, capsules, or sachets for two to four weeks. *Lactobacillus reuteri* as a single-strain probiotic was the focus of one investigation.

Probiotic supplementation has shown positive impacts on salivary function and dental health outcomes in multiple trials. V Manek et al. (2025) found that among healthy adults, plaque pH significantly increased and gingival inflammation decreased (V Manek et al., 2025). In a similar vein, Pereira Riveros et al. (2025) found that patients with head and neck cancer had better stimulated salivary flow and a lower total bacterial load, including Fusobacterium nucleatum (Pereira Riveros et al., 2025). Additionally, Alamoudi et al. (2018) discovered that preschoolers’ salivary buffering capacity improved and Streptococcus mutans numbers significantly decreased (Alamoudi et al., 2018). Widyarman et al. (2022) demonstrated short-term modulation of the salivary microbiome following probiotic administration; however, these effects were not sustained after cessation of the intervention (Widyarman et al., 2022). Probiotics have been demonstrated to improve microbial balance and lower harmful bacterium populations in the oral and gut microbiota. Studies by He et al. (2022) and Zheng et al. (2023) found improvements in gastrointestinal outcomes, such as decreased gastrointestinal unpleasant effects, better bowel habits, and favorable regulation of immunological and inflammatory markers, in addition to oral microbial alterations. Overall, the results indicate that probiotic supplementation may have a positive impact on gut health, salivary parameters, and oral microbiota. However, there was variation in probiotic strains, dosage schedules, length of intervention, and outcome measures among the studies, which limited the direct comparability of the findings (He et al., 2022; Zheng et al., 2023).

### Risk of bias evaluation

The RoB 2.0 method was used to assess the risk of bias in the included randomized controlled trials, and the results showed an overall moderate quality of evidence. Strong methodological rigor was indicated by studies by V Manek P et al. and Widyaraman AS et al. that showed an overall low risk of bias across most areas (Widyarman et al., 2022; V Manek et al., 2025). In a similar vein, AlamoudiNM et al. demonstrated primarily minimal risk with just minor issues during the randomization procedure (Alamoudi et al., 2018). Studies like Pereira Riveros T et al. and Zhang Y et al., on the other hand, raised certain issues, especially in areas pertaining to the randomization procedure and departures from planned interventions (Zheng et al., 2023; Pereira Riveros et al., 2025]. He C et al. overall quality assessment was affected by a higher risk of bias, mostly because of missing outcome data and problems with departures from targeted interventions (He et al., 2022).

While missing outcome data appeared as a major source of high risk in some research, the domains of outcome assessment and the selection of reported outcomes consistently showed minimal risk of bias across investigations. Overall, even though the majority of research were classified as low risk or raising some concerns, the existence of high-risk domains in a small number of studies calls for careful interpretation of the results (shown in **Figure 2**).

### Certainty of evidence

The well-designed multicenter double-blind randomized controlled trial by He C et al. (2022) revealed high-certainty evidence (He et al., 2022). This study provided strong evidence of probiotics’ capacity to reduce gastrointestinal distress and stabilize microbiota with few methodological errors. Studies by Pereira Riveros T et al. (2025) and Alamoudi NM et al. (2018) reported moderate-certainty evidence (Alamoudi et al., 2018; Pereira Riveros et al., 2025). These randomized controlled trials showed how probiotics can improve salivary flow and microbiological parameters while lowering cariogenic bacteria. However, due to imprecision, which was mostly caused by shorter follow-up periods and comparatively smaller sample sizes, the certainty was reduced.

Low-certainty evidence was identified in studies by Widyarman AS et al. (2022),V Manek P et al. (2025), and Zheng Y et al. (2023) (Widyarman et al., 2022; Zheng et al., 2023; V Manek et al., 2025). Methodological issues such small sample numbers, the absence of control groups in certain studies, brief intervention durations, and indirect outcomes were the primary causes of the decrease in certainty. The conclusions’ strength and generalizability are constrained by these considerations.

Overall, despite the fact that probiotics seem to improve gingival health, oral microbiota, and associated clinical indicators, there is still a lack of confidence in these results because of variations in study designs, populations, and end measures. Therefore, additional large-scale, well-designed randomized controlled studies with extended follow-up periods are needed to increase the certainty of the evidence and support decisive clinical recommendations, even though the data points to good treatment potential (further details please see **Table 2**).

## Discussion

Current evidence suggests that probiotic supplementation can modify the composition of the oral microbiota (lower cariogenic and certain periodontal pathogens) as well as salivary biochemical markers (buffer capacity, salivary cytokines) in a strain- and dose-dependent way. Salivary flow is not always affected. Changes in gut microbiota after probiotic treatment are well-established and probably contribute to some of the systemic immunological advantages that are occasionally noted.

Probiotics have a well-established effect on the composition of the gut microbiota, and there is strong evidence that they can improve intestinal barrier integrity, regulate immune function, and restore microbial equilibrium. These gut-mediated effects, such as decreased systemic inflammation and enhanced immunological control, are becoming more widely acknowledged as possible contributions to systemic health benefits. Within this context, the concept of the oral–gut axis has gained prominence, suggesting a bidirectional relationship between oral and intestinal microbial communities.

Probiotic-induced alterations in gut microbiota may influence oral health indirectly through systemic pathways, including immune modulation, production of short-chain fatty acids, and regulation of inflammatory signaling (V Manek et al., 2025).These mechanisms may help explain the observed improvements in oral inflammatory status and salivary biomarkers in researches (Pereira Riveros et al., 2025). Studies suggest that systemic immunomodulatory effects induced by probiotics—such as reduced inflammatory mediators and improved mucosal immunity—may contribute to observed changes in oral inflammatory status and salivary biomarkers (Zheng et al., 2023). However, the extent and clinical significance of these interconnected effects remain incompletely understood. Further well-designed, large-scale randomized controlled trials with standardized protocols are essential to establish definitive clinical recommendations and to better elucidate the mechanisms underlying probiotic action within the oral–gut axis.

### Strengths and limitations of the evidence

The study’s strengths include the inclusion of several systematic reviews and randomized controlled trials, which provide a reasonable level of clinical evidence. Furthermore, the suggested mechanisms of action such as the synthesis of antimicrobial metabolites and the modification of the oral and gut microbiome are biologically conceivable and lend credence to the potential therapeutic use of probiotics. The analysis is constrained, nonetheless, by the very small number of included trials and the considerable variation in probiotic strains, doses, delivery methods (such as lozenges, dairy products, capsules), and intervention durations. Additionally, a large number of the included studies have small sample numbers, brief follow-up periods, and inconsistent outcome measures, especially when it comes to microbial detection and salivary measurement methodologies. Additionally, the effects of probiotic supplementation seem to be temporary, frequently waning after stopping. This is probably because probiotics have a limited capacity to establish long-term colonization in the oral cavity without constant use.

### Clinical implications

For people who are at high risk of oral disorders including gingivitis and dental caries, probiotics may be used as short-term, supplementary treatments, especially if there is evidence to support particular strains (such Lactobacillus reuteri). However, they should be used as supportive adjuncts rather than as a replacement for well-established preventive interventions, such as fluoride-based methods and mechanical plaque reduction. There is also further proof that probiotics are beneficial for some gastrointestinal illnesses, such as diarrhea brought on by antibiotics and some inflammatory ailments. To guarantee the best possible therapeutic results, however, clinical advice should continue to be strain-, dose-, and indication-specific.

### Research gaps and future directions

Standardized oral and systemic outcome measures should be used in well-designed head-to-head randomized controlled studies that compare various probiotic strains, doses, administration methods, and intervention durations. To assess the sustainability of probiotic benefits and colonization dynamics, longer follow-up times are required. To better understand the oral–gut axis, mechanistic investigations combining alterations in the oral microbiome with gut microbial and metabolomic profiles are crucial. Standardized outcome measures, such as consistent procedures for microbiological quantification, clinical indices, and salivary measurement, would further improve study comparability and enable reliable meta-analyses.

## Conclusion

Thus, it was seen that current studies suggested that probiotic administration may enhance the composition of the oral microbiota and a number of salivary biochemical parameters, particularly with regard to reducing cariogenic and certain periodontal infections and altering inflammatory markers. These effects appear to be strain-specific and dose-dependent, albeit data from several studies are not entirely consistent.

There is also conflicting evidence about how probiotics affect salivary flow, which emphasizes the need for more research. Although probiotics have a well-established role in regulating gut microbiota, their indirect effects on oral health through systemic routes, such as the suggested oral–gut axis, are still poorly understood and should be interpreted cautiously.

## Data Availability

The original contributions presented in the study are included in the article/supplementary material. Further inquiries can be directed to the corresponding authors.
